# Identifying demographic, social and clinical predictors of biologic therapy effectiveness in psoriasis: a multicentre longitudinal cohort study

**DOI:** 10.1111/bjd.16776

**Published:** 2018-08-28

**Authors:** R.B. Warren, A. Marsden, B. Tomenson, K.J. Mason, M.M. Soliman, A.D. Burden, N.J. Reynolds, D. Stocken, R. Emsley, C.E.M. Griffiths, C. Smith, the PSORT Consortium and on behalf of the BADBIR Study Group

**Affiliations:** ^1^ Dermatology Centre Salford Royal NHS Foundation Trust The University of Manchester Manchester Academic Health Science Centre NIHR Manchester Biomedical Research Centre Manchester U.K.; ^2^ Centre for Biostatistics School of Health Sciences The University of Manchester Manchester Academic Health Science Centre Manchester U.K.; ^3^ Division of Musculoskeletal and Dermatological Sciences The University of Manchester Manchester U.K.; ^4^ Department of Pharmacy Practice Faculty of Pharmacy Mansoura University Mansoura Egypt; ^5^ Department of Dermatology Royal Infirmary of Edinburgh Edinburgh U.K.; ^6^ Dermatological Sciences, Institute of Cellular Medicine, Medical School Newcastle University, NIHR Newcastle Biomedical Research Centre and Department of Dermatology, Royal Victoria Infirmary, Newcastle Hospitals NHS Foundation Trust Newcastle upon Tyne U.K.; ^7^ Clinical Trials Research Unit Leeds Institute of Clinical Trials Research University of Leeds Leeds U.K.; ^8^ Institute of Psychiatry, Psychology and Neuroscience King's College London London U.K.; ^9^ St John's Institute of Dermatology Guy's and St Thomas’ NHS Foundation Trust London U.K.

## Abstract

**Background:**

Biologic therapies have revolutionized the treatment of moderate‐to‐severe psoriasis. However, for reasons largely unknown, many patients do not respond or lose response to these drugs.

**Objectives:**

To evaluate demographic, social and clinical factors that could be used to predict effectiveness and stratify response to biologic therapies in psoriasis.

**Methods:**

Using a multicentre, observational, prospective pharmacovigilance study (BADBIR), we identified biologic‐naive patients starting biologics with outcome data at 6 (*n* = 3079) and 12 (*n* = 3110) months. Associations between 31 putative predictors and outcomes were investigated in univariate and multivariable regression analyses. Potential stratifiers of treatment response were investigated with statistical interactions.

**Results:**

Eight factors associated with reduced odds of achieving ≥ 90% improvement in Psoriasis Area and Severity Index (PASI 90) at 6 months were identified (described as odds ratio and 95% confidence interval): demographic (female sex, 0·78, 0·66–0·93); social (unemployment, 0·67, 0·45–0·99); unemployment due to ill health (0·62, 0·48–0·82); ex‐ and current smoking (0·81, 0·66–0·99 and 0·79, 0·63–0·99, respectively); clinical factors (high weight, 0·99, 0·99–0·99); psoriasis of the palms and/or soles (0·75, 0·61–0·91); and presence of small plaques only compared with small and large plaques (0·78, 0·62–0·96). White ethnicity (1·48, 1·12–1·97) and higher baseline PASI (1·04, 1·03–1·04) were associated with increased odds of achieving PASI 90. The findings were largely consistent at 12 months. There was little evidence for predictors of differential treatment response.

**Conclusions:**

Psoriasis phenotype and potentially modifiable factors are associated with poor outcomes with biologics, underscoring the need for lifestyle management. Effect sizes suggest that these factors alone cannot inform treatment selection.

## Abstract 2

**What's already known about this topic?**

- Biologic therapy used in the treatment of moderate‐to‐severe psoriasis differs in its effectiveness across patients.
- Previous research has indicated that patients with a higher body mass index, who smoke or who have smoked, and with a lower baseline Psoriasis Area and Severity Index (PASI) are less likely to have a good outcome with biologic therapy for the treatment of moderate‐to‐severe psoriasis.

**What does this study add?**

- This large‐scale study in a real‐world setting confirms that weight, smoking status and baseline PASI are associated with effectiveness of biologic therapy.
- There is evidence that non‐white ethnicity, female sex, unemployment, psoriasis of the palms and soles and the presence of small chronic plaques are associated with poor outcomes with biologics.
- There is some evidence that men have a comparatively worse response to etanercept, relative to adalimumab, than women. Otherwise, most factors do not appear to be predictors of differential treatment response.

Respond to this article

Psoriasis is a common chronic inflammatory skin condition, which is recognized by the World Health Organization as a serious noncommunicable disease that requires effective management.^1^ Moderate‐to‐severe psoriasis (approximately > 10% body surface area involvement) is increasingly treated with biologic therapies. Clinical trials indicate that biologics are effective for many patients in the short term,^2^ but not all patients respond and, importantly, registry data show that ≥ 15% of patients discontinue treatment per annum.^3^ In addition, biologics are expensive (in the U.K. generally > £10 000 per annum), so nonresponse or loss of response is costly.

Significant investment into investigation of genomic factors that might influence treatment outcomes has not yet made a clinical impact, as exemplified by a recent large‐scale investigation in patients with rheumatoid arthritis, which concluded that genetic variants alone would be unable to predict response to anti‐tumour necrosis factor (TNF)‐α therapies.^4^ Indeed, the factors linked to lack of effectiveness of biologic drugs are varied, but on the whole are poorly understood, aside from high bodyweight, which has consistently been associated with worse outcomes for most biologic therapies.^5^, ^6^, ^7^, ^8^, ^9^ This raises the possibility that other, as yet unidentified, demographic, social or clinical factors may be relevant in determining response. If this is the case, these would not be costly to ascertain and could be modifiable. Investigations assessing the role that these factors may play in predicting treatment outcomes in psoriasis have been neglected.

The British Association of Dermatologists Biologic Interventions Register (BADBIR) is a U.K.‐ and Republic of Ireland‐based, multicentre, pharmacovigilance registry for adults with psoriasis starting on biologic (and standard systemic) therapies, which was initiated in September 2007.^10^ This longitudinal cohort dataset includes detailed demographic, social and clinical information on all participants and thus provides a resource of sufficient size and detail to investigate comprehensively the extent to which these factors predict treatment outcomes. The specific aims of the current study were: (i) to identify baseline demographic, social and clinical factors that predict the effectiveness of adalimumab, etanercept and ustekinumab therapy; (ii) to provide clinically useful measures of association between predictors and outcome; and (iii) to investigate effect modification of factors by treatment type and consequentially to identify factors that could be used to inform treatment stratification.

## Materials and methods

### Study design, setting and participants

An observational cohort study was performed using data from BADBIR.^10^ Patients with chronic plaque psoriasis enrolled in BADBIR up to 1 February 2017 who initiated either adalimumab, etanercept or ustekinumab as a first‐line biologic formed the study population for this analysis. Infliximab was not included, as the baseline severity criteria to start this drug are different from those of other biologics and the cohort size is considerably smaller, creating difficulties for interaction analyses. Both a baseline Psoriasis Area Severity Index (PASI) and follow‐up PASI (at approximately 6 or 12 months) were required for inclusion in the study. For the main analyses, patients were included regardless of whether or not they were still receiving the treatment at the time of the follow‐up PASI, as stopping therapy may be linked to poor response. Patients who had withdrawn from therapy at the time of the outcome measure were excluded in a sensitivity analysis (Tables S1 and S2; see Supporting Information).

### Outcome assessment

The primary outcome was a binary indicator of a reduction of PASI by ≥ 90% (PASI 90) within 6 months following treatment initiation. A 4–8‐month window was chosen to approximate 6 months. If a patient had multiple PASIs within this time frame, the lowest value was used. Baseline PASI was defined as the nearest PASI up to and including 6 months prior to the treatment start date.^3^, ^11^ PASI 90 at 12 months (defined as a 10–14‐month window) was also investigated, as were PASI 75 and the attainment of absolute PASI ≤ 1·5; the latter was chosen to approximate PASI 90 without relying on the baseline PASI.

### Covariates

Demographic factors (age at biologic initiation, sex, ethnicity as white or non‐white), lifestyle and social factors [work status, body mass index (BMI), weight, alcohol intake, smoking status as never/ex/current], skin type, disease characteristics (baseline PASI; disease duration; family history; flexural psoriasis; psoriasis involvement on the scalp, nails and palms and/or soles; small/large chronic plaque; psoriatic arthritis), Fitzpatrick skin type, comorbidities (hypertension, angina, heart attack, stroke, latent tuberculosis, diabetes, depression, dyslipidaemia, nonskin cancer) and cotherapy factors (previous use of conventional drugs, baseline concomitant methotrexate or ciclosporin) were assessed for associations with PASI outcomes. The variables were chosen due to their availability in BADBIR.

### Statistical analyses

Univariate analyses (*t*‐tests for approximately normally distributed continuous variables, Mann–Whitney test for non‐normal continuous variables, χ^2^‐tests for binary or categorical variables) were performed to identify baseline factors associated with PASI 90, PASI 75 and absolute PASI ≤ 1·5 within 6 months and 12 months following the initiation of treatment.

Logistic multivariable regression models were fitted to assess variables collectively with *P* < 0·2 from the corresponding univariate analysis. The inclusion of those with *P* > 0·2 was also explored to see whether any of these had an association with the outcome when adjusting for other covariates. Predictors with *P* < 0·05 in the 6‐month analysis of PASI 90 were also included in the subsequent models for other outcomes. Any biologic therapy received was included in each multivariable model to ensure that associations between the covariates and the outcome were independent of choice of therapy. Continuous variables were included as such in the main analyses, but additional models were fitted where they were categorized to aid interpretation.

Calibration (goodness of fit) of each model was assessed by calculating the model‐predicted probability of the outcome for each individual and assessing the percentage of people who did observe the outcome in categories of the predicted probabilities and by using the Hosmer–Lemeshow test.^12^ For example, we would expect around 20% of people who had a model‐predicted probability of 0·2 to have observed the outcome. Discrimination (predictive ability) of the model was assessed by calculating the area under the receiver operating curve.^12^

Risk differences and the corresponding number needed to treat (NNT) were calculated for each variable included in the multivariable analysis for PASI 90 at 6 months to give a more interpretable indication of the effect sizes.^13^ Adjusted risk differences were estimated from the output of the logistic model by averaging over the effects of the other model covariates.^14^ The NNT is the reciprocal of the risk difference. Continuous variables were dichotomized by the sample median for this analysis due to requirements of the methodology.

Interactions between the specific biologic therapy and the variables included in the multivariable regression for PASI 90 at 6 months were investigated to identify effect modification of these variables by treatment type. Interaction was assessed on the multiplicative scale via the ratio of odds ratios (ORs) and on the additive scale via the difference in risk differences. A comparison of absolute treatment effects (here, risk differences) is more appropriate for the investigation of differential treatment response as it is not influenced by differences in baseline risks of the outcome across the subgroups being compared, unlike comparisons of relative treatment measures.^15^ Each interaction analysis was adjusted for all other variables included in the PASI 90 6‐month multivariate model.

Where present, missing data for potential predictor variables were accounted for by multiple imputation, performed using the Stata chained command with 20 imputed datasets based on the full set of independent variables with complete data and the dependent variable. Analyses were performed in Stata 14.^16^

### Sensitivity analyses

The multivariable analyses for PASI 90 at 6 and 12 months were repeated: (i) excluding patients who were no longer receiving the prescribed first‐line biologic at the time of the 6‐month PASI; and (ii) incorporating sampling weights (defined as the probability of being included in the sample) to account for potential sampling bias due to the exclusion of patients without the required PASIs.^17^

## Results

### Participants, exposure, baseline characteristics and response rates

**Figure 1:**
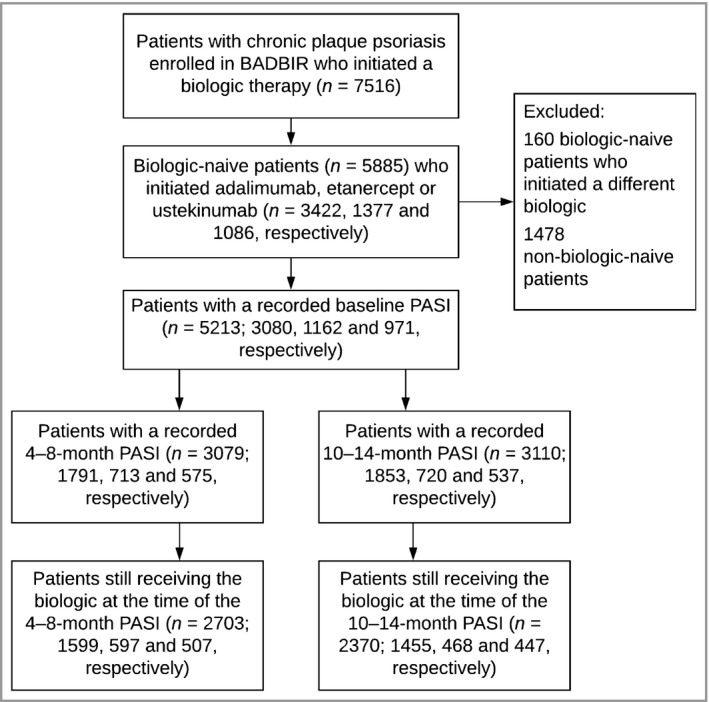
Flow diagram showing selection of patients for inclusion in the main analyses. This figure shows how patients in the British Association of Dermatologists Biologic Interventions Register (BADBIR) were selected to contribute to the study. PASI, Psoriasis Area and Severity Index.

Data from 7516 patients with chronic plaque psoriasis enrolled in BADBIR up to 1 February 2017 were available (Fig. 1). Of these, 5885 biologic‐naive patients started adalimumab (3422, 58·1%), etanercept (1377, 23·4%) or ustekinumab (1086, 18·5%). Furthermore, 3079 (52·3%) had a PASI score at baseline and 6 months and 3110 (52·8%) had a PASI at baseline and 12 months. The median number of days between the baseline PASI and the treatment start date was 27 days, and 90% of patients’ baseline PASI scores were within the 90 days prior. Their baseline characteristics were in line with those in previous reports.^18^ The level of missingness for most variables was < 10%, but it was high for alcohol intake (around 36%) (Table S3; see Supporting Information).

Of those starting adalimumab, etanercept and ustekinumab, 50·1%, 20·1% and 49·9%, respectively, achieved PASI 90 at 6 months, with comparable outcomes at 12 months (for PASI 75 and PASI ≤ 1·5 outcomes, see Table S4 in Supporting Information).

### Characterization of demographic, social and clinical factors associated with response

The univariate analyses assessing the association between each variable and the attainment of PASI 90 (Table S5; see Supporting Information) identified 16 variables with *P* < 0·2 that were taken forward to the multivariable analysis. White ethnicity was also taken forward, as subsequent exploration indicated that this had a significant association when other covariates were adjusted for. BMI was excluded due to high correlation with weight.

**Figure 2:**
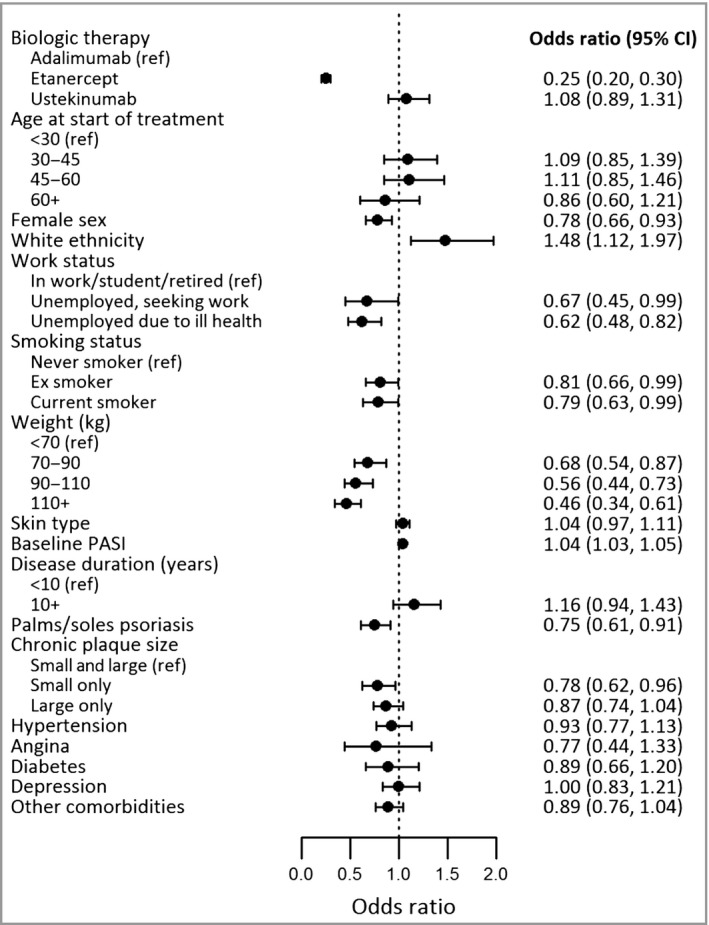
This figure presents the odds ratios and corresponding 95% confidence intervals (CIs) investigating the associations between baseline factors and the attainment of ≥ 90% improvement in Psoriasis Area and Severity Index (PASI) at 6 months. Estimates were obtained from a multivariable logistic regression analysis. Results for white ethnicity and skin type are estimated from a model excluding the other due to similarities in these two variables. Skin type was included as a continuous variable, where a higher value represents darker skin.

Of the demographic and social factors, female sex [OR 0·78, 95% confidence interval (CI) 0·66–0·93], unemployment in those seeking work (OR 0·67, 95% CI 0·45–0·99), unemployment due to ill health (OR 0·62, 95% CI 0·48–0·82) and both ex‐smoking (OR 0·81, 95% CI 0·66–0·99) and current smoking (OR 0·79, 95% CI 0·63–0·99) were associated with reduced odds of achieving PASI 90 at 6 months following treatment initiation (Fig. 2 and Table S6; see Supporting Information). White ethnicity (OR 1·48, 95% CI 1·12–1·97) was associated with increased odds of achieving PASI 90 at 6 months.

With respect to the clinical factors, higher weight (OR 0·99, 95% CI 0·99–0·99), psoriasis involving the palms and/or soles (OR 0·75, 95% CI 0·61–0·91) and having only small chronic plaques (compared with both small and large chronic plaques) (OR 0·78, 95% CI 0·62–0·96) were associated with reduced odds of achieving PASI 90 at 6 months. A higher baseline PASI was associated with increased odds of this outcome (OR 1·04, 95% CI 1·03–1·05). Furthermore, categorization of weight showed that those weighing 70–90 kg, 90–110 kg and ≥ 110 kg had lower odds of achieving PASI 90 at 6 months than those weighing < 70 kg (OR 0·68, 95% CI 0·54–0·87; OR 0·56, 95% CI 0·44–0·73 and OR 0·46, 95% CI 0·34–0·61, respectively).

The model fit the data well (Hosmer–Lemeshow *P*‐value 0·61) and had an area under the curve of 0·70 (95% CI 0·67–0·71), demonstrating reasonably good predictive ability (Table S7; see Supporting Information).

Findings for the multivariable analysis for predictors of PASI 90 at 12 months were consistent with those at 6 months regarding sex, weight, ex‐smoking and psoriasis of the palms and/soles (Fig. S1 and Table S6; see Supporting Information), but there was less evidence for an association with work status, current smoking and plaque size. However, there was evidence that nail psoriasis was associated with reduced odds of achieving PASI 90 at 12 months (OR 0·85, 95% CI 0·73–1·00) and that psoriatic arthritis was associated with increased odds of this outcome (OR 1·22, 95% CI 1·01–1·47). The calibration and discrimination assessment statistics were similar to those at 6 months.

Risk differences and corresponding NNTs aid interpretation regarding the clinical utility of the potential predictors in the PASI 90 analysis (Table S8; see Supporting Information). For example, patients who had a bodyweight > 90 kg were, in absolute terms, around 7·6% less likely to achieve PASI 90 at 6 months than those with a weight < 90 kg, and current and ex‐smokers were estimated to be, respectively, 5·5% and 5·0% less likely to achieve PASI 90 at 6 months than nonsmokers.

The first sensitivity analysis that excluded patients who had withdrawn from therapy by the time of their 6‐ or 12‐month PASI evaluation (giving *n* = 2703 and *n* = 2370 at 6 and 12 months, respectively) gave similar results to the main analyses (Tables S1 and S2; see Supporting Information). However, the association between female sex and outcome at 12 months was weaker, suggesting that the negative association in the main analysis was partly due to more women withdrawing from therapy and subsequently not achieving a good PASI reduction. Incorporating sampling weights to account for potential sampling bias from excluding patients without the required PASI scores also gave similar results to the main analyses (Tables S1 and S2).

Results for the analyses exploring predictors of PASI 75 and absolute PASI ≤ 1·5 are shown in Tables S9 and S10 (see Supporting Information).

### Predictors of differential treatment response

The majority of variables tested were not associated with differential response to etanercept or ustekinumab compared with adalimumab (Table S11; see Supporting Information). However, there was a suggestion that the absolute effect of etanercept compared with adalimumab was more negative in men than in women (difference in risk differences 0·079, 95% CI 0·002–0·16). In other words, male patients had a comparatively worse response to etanercept, relative to adalimumab, than female patients.

## Discussion

In this large‐scale, real‐world dataset of patients with psoriasis we have found evidence that female sex, non‐white ethnicity, smoking (current or ever), higher weight, unemployment and psoriasis involving the palms and/or soles are associated with a reduced likelihood of response to biologic therapy up to 1 year. We also provided clinically useful measures of the associations between predictors and outcomes via risk differences and NNTs. In addition, a key finding from this study is that, while there are factors associated with a poorer or better response to biologic therapy for chronic plaque psoriasis in general, most factors do not appear to be predictors of treatment stratification: they would not aid the selection of the optimal biologic therapy for a patient out of the available options.

To our knowledge, other studies have found neither female sex nor non‐white ethnicity to be negatively associated with response to biologics in psoriasis.^5^ Female sex has previously been shown to be associated with discontinuation of biologic therapy in patients with psoriasis, but this was mainly attributed to adverse events.^3^

This study shows that people who have plaque psoriasis affecting their palms and/or soles have a lower PASI response at both 6 and 12 months. There are relatively few clinical data on this subtype of psoriasis; of the drugs studied in this analysis only adalimumab has been the subject of a formal, randomized controlled trial. This showed that 31% of patients with psoriasis of the hands or feet (which we assume to be similar to psoriasis of the palms or soles) achieved a clear or nearly clear response on a Physician's Global Assessment scale, compared with 3% on placebo.^19^ This response is considerably lower than that seen for psoriasis overall, where responses of > 60% achieving PASI 75 would be expected. It should be noted that this study was not looking at overall response of disease and focused solely on response of the hands and feet. Effective treatments for psoriasis at these sites remain a priority area for research.

Our findings agree with others that the modifiable factors of higher weight and current smoking are associated with a poorer response to first‐line biologic therapy.^6^, ^8^ Other studies have mainly analysed BMI, rather than weight, but we consider there to be a significant correlation between the two. The association was weaker for ex‐smokers than for current smokers.

A higher baseline PASI was associated with an increased likelihood of achieving both PASI 90 and PASI 75, but not absolute PASI ≤ 1·5. This suggests that patients with higher baseline PASI have on average a larger relative reduction in PASI score than those with a lower baseline PASI score, but do not achieve a lower absolute PASI score. This association may arise because PASI 75 and PASI 90 are functions of baseline PASI, and achieving these outcomes requires patients with a lower baseline PASI to reach a lower follow‐up PASI than those with a higher baseline PASI.

Some findings are consistent with those in other disease areas. Female sex has been found to be associated with a poor response to anti‐TNF therapies in rheumatoid arthritis (RA)^20^, ^21^, ^22^ and ankylosing spondylitis.^23^ It is not clear why women have a poorer response to these therapies, but one factor may be that, in general, women taking a variety of therapies have a higher rate of severe adverse events.^24^, ^25^ Smoking has been found to be associated with poor response to anti‐TNF therapies in RA^20^, ^21^ and infliximab in Crohn disease.^26^, ^27^ Studies have found a negative association between BMI and response to anti‐TNF therapies in RA^21^, ^22^ and ankylosing spondylitis.^23^

This study has a number of strengths: the prospective cohort study design, the large sample size, fully industry‐independent data analysis, and the external validity of the results due to the participation of multiple dermatology centres (153) across the U.K. and the Republic of Ireland.

In terms of the methodology, performing multivariable analyses in addition to univariate analyses allows a more confident interpretation regarding the independent association between the predictors and the PASI outcomes. For example, several comorbidities that were associated with the outcome in the univariate analyses had attenuated associations when other variables such as weight and smoking were accounted for in the multivariable analyses. Another advantage of our methodology is that we investigated differential response to the separate biologic therapies. Most studies investigate only predictors of a good outcome under treatment, but predictors of differential response aid clinicians in choosing the best therapy for each patient. We also produced clinically interpretable effect sizes of the associations.

A significant limitation of our study is that only about half of the patients registered with BADBIR who initiated one of the biologics investigated had PASI measurements at the times required for this analysis, leading to a high number of excluded patients. Assessing absolute PASI response overcomes the need for a baseline PASI, but most patients were excluded due to having no follow‐up PASI (86·2% and 87·4% of those excluded at 6 and 12 months, respectively). However, we did not find a notable difference in the baseline characteristics of those with and without the required PASIs, and performing inverse probability weighting to account for unrepresentative missingness (Tables S1 and S2; see Supporting Information) gave similar results to the main analysis. The missingness of follow‐up PASIs would be problematic if those with a missing PASI had typically higher or lower PASI than those with the required PASI scores. However, the overall levels of attainment of PASI 90 and PASI 75 at 6 and 12 months were as expected, suggesting that this may not be the case.

We recognize that, due to the wealth of information collected in BADBIR, several statistical tests were performed, increasing the risk of type I errors. We can have more confidence that the findings in line with previous research (weight, smoking status, baseline PASI, psoriasis of the palms and soles) are not spurious. We would be interested to see whether the findings relating to white ethnicity, work status, small chronic plaques and sex can be replicated independently.

In conclusion, data from more than 3000 patients in BADBIR indicate that high bodyweight, smoking status, psoriasis on the palms and/or soles, female sex, non‐white ethnicity and unemployment are associated with poorer effectiveness of biologic therapies up to 1 year following initiation. Some of these factors are modifiable and confirm the important role that a dermatologist plays in educating patients about behaviour change and lifestyle management in addition to targeted biologic therapy. Counselling regarding smoking cessation and weight‐loss programmes are an important part of general health management; this study highlights how these factors detrimentally affect treatment effectiveness.^28^, ^29^ Furthermore, the study provides some of the first real‐world data to support the hypothesis that patients with plaque disease of the palms and/or soles do not achieve the same levels of overall response as those without involvement. We found little evidence that the clinical variables were associated with differential response to the separate biologics, therefore it is unlikely we can produce a reliable tool to select the optimal therapy based on these factors alone. It is possible that an integrated approach across omics and clinical data will be needed.^4^

## Supporting information


**Fig S1.** Forest plot of odds ratios estimated in the multivariable analysis investigating associations between baseline factors and the attainment of ≥ 90% improvement in Psoriasis Area and Severity Index at 12 months.

**Table S1.** Results of sensitivity analyses investigating predictors of ≥ 90% improvement in Psoriasis Area and Severity Index at 6 months.
**Table S2.** Results of sensitivity analyses investigating predictors of ≥ 90% improvement in Psoriasis Area and Severity Index at 12 months.
**Table S3.** The baseline characteristics of the biologic‐naive cohort.
**Table S4.** Response rates for ≥ 90% and ≥ 75% improvement in Psoriasis Area and Severity Index (PASI) and absolute PASI ≤ 1·5 at 6 and 12 months.
**Table S5.** Results of the univariate analyses exploring associations between potential risk factors and the attainment of ≥ 90% improvement in Psoriasis Area and Severity Index at 6 months in patients who initiated adalimumab, etanercept or ustekinumab.
**Table S6.** Results of multivariable logistic regression analyses investigating predictors of ≥ 90% improvement in Psoriasis Area and Severity Index at both 6 and 12 months in patients who initiated adalimumab, etanercept or ustekinumab.
**Table S7.** Calibration and discrimination analysis statistics for the multivariable regression models.
**Table S8.** Estimated risk differences and numbers needed to treat for each predictor included in the multivariable analysis on the attainment of ≥ 90% improvement in Psoriasis Area and Severity Index at 6 months in patients who initiated adalimumab, etanercept or ustekinumab.
**Table S9.** Results of multivariable logistic regression analyses investigating predictors of ≥ 75% improvement in Psoriasis Area and Severity Index at both 6 and 12 months in patients who initiated adalimumab, etanercept or ustekinumab.
**Table S10.** Results of multivariable logistic regression analyses investigating predictors of absolute Psoriasis Area and Severity Index 1·5 at both 6 and 12 months in patients who initiated adalimumab, etanercept or ustekinumab.
**Table S11.** Results of interaction analyses investigating differential relative and absolute effects of each predictor on the attainment of ≥ 90% improvement in Psoriasis Area and Severity Index at 6 months across patients who initiated adalimumab, etanercept or ustekinumab.

**Video S1** Author video.
