# Supplementary figures and images for: Identifying demographic, social and clinical predictors of biologic therapy effectiveness in psoriasis: a multicentre longitudinal cohort study
Source: Br J Dermatol. 2018 Aug 28;180(5):1069–76. doi: 10.1111/bjd.16776 (PMC6519065; doi:10.1111/bjd.16776)

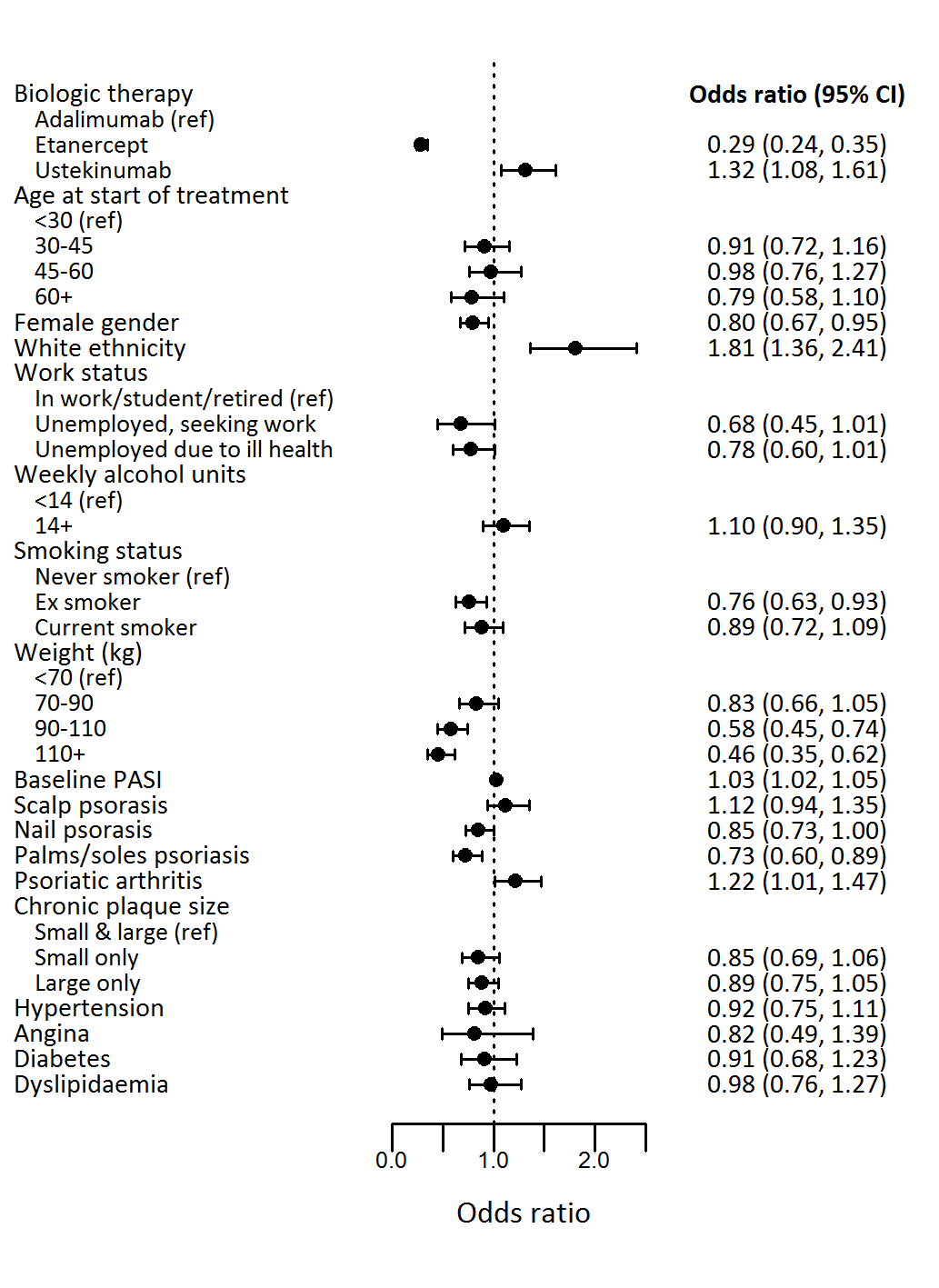

Supplement: Supplementary file 1 — Fig S1. Forest plot of odds ratios estimated in the multivariable analysis investigating associations between baseline factors and the attainment of ≥ 90% improvement in Psoriasis Area and Severity Index at 12 months. [file BJD-180-1069-s001.tiff]
