# Supplementary material for: Identifying demographic, social and clinical predictors of biologic therapy effectiveness in psoriasis: a multicentre longitudinal cohort study
Source: Br J Dermatol. 2018 Aug 28;180(5):1069–76. doi: 10.1111/bjd.16776 (PMC6519065; doi:10.1111/bjd.16776)
Supplement: Supplementary file 2 — Table S1. Results of sensitivity analyses investigating predictors of ≥ 90% improvement in Psoriasis Area and Severity Index at 6 months. Table S2. Results of sensitivity analyses investigating predictors of ≥ 90% improvement in Psoriasis Area and Severity Index at 12 months. Table S3. The baseline characteristics of the biologic‐naive cohort. Table S4. Response rates for ≥ 90% and ≥ 75% improvement in Psoriasis Area and Severity Index (PASI) and absolute PASI ≤ 1·5 at 6 and 12 months. Table S5. Results of the univariate analyses exploring associations between potential risk factors and the attainment of ≥ 90% improvement in Psoriasis Area and Severity Index at 6 months in patients who initiated adalimumab, etanercept or ustekinumab. Table S6. Results of multivariable logistic regression analyses investigating predictors of ≥ 90% improvement in Psoriasis Area and Severity Index at both 6 and 12 months in patients who initiated adalimumab, etanercept or ustekinumab. Table S7. Calibration and discrimination analysis statistics for the multivariable regression models. Table S8. Estimated risk differences and numbers needed to treat for each predictor included in the multivariable analysis on the attainment of ≥ 90% improvement in Psoriasis Area and Severity Index at 6 months in patients who initiated adalimumab, etanercept or ustekinumab. Table S9. Results of multivariable logistic regression analyses investigating predictors of ≥ 75% improvement in Psoriasis Area and Severity Index at both 6 and 12 months in patients who initiated adalimumab, etanercept or ustekinumab. Table S10. Results of multivariable logistic regression analyses investigating predictors of absolute Psoriasis Area and Severity Index 1·5 at both 6 and 12 months in patients who initiated adalimumab, etanercept or ustekinumab. Table S11. Results of interaction analyses investigating differential relative and absolute effects of each predictor on the attainment of ≥ 90% improvement in Psoriasis Area an [file BJD-180-1069-s002.docx]

Full legend for Figure S1

**Fig S1.** Forest plot of odds ratios estimated in the multivariable analysis investigating associations between baseline factors and the attainment of PASI 90 at 12 months.

This figure presents the odds ratios and corresponding 95% confidence intervals investigating the associations between baseline factors and the attainment of PASI 90 at 12 months. Estimates were obtained from a multivariable logistic regression analysis.

**Table S1** Results of sensitivity analyses investigating predictors of PASI 90 at 6 months in patients who initiated adalimumab, etanercept or ustekinumab when 1) patients who withdrew from treatment before the PASI measure were excluded and 2) weighting to account for sampling was applied

|  | **Sensitivity analysis 1** | | **Sensitivity analysis 2** | |
| --- | --- | --- | --- | --- |
| **Predictor** | **Odds ratio (95% CI)** | ***p*-value** | **Odds ratio (95% CI)** | ***p*-value** |
| Biologic therapy  Adalimumab (reference group)  Etanercept  Ustekinumab | 0.23 (0.18, 0.29)  0.98 (0.80, 1.21) | <0.001  0.89 | 0.24 (0.20, 0.30)  1.10 (0.90, 1.34) | <0.001  0.36 |
| Age in years at start of treatment | 1.003 (0.995, 1.011) | 0.44 | 1.00 (0.994, 1.001) | 0.68 |
| Female gender | 0.80 (0.67, 0.96) | 0.014 | 0.79 (0.67, 0.94) | 0.006 |
| White ethnicity^1^ | 1.46 (1.08, 1.97) | 0.013 | 1.47 (1.11, 1.97) | 0.008 |
| Work status  In work/student/retired (reference  group)  Unemployed and seeking work  Unemployed due to ill health | 0.70 (0.46, 1.06)  0.69 (0.52, 0.93) | 0.091  0.013 | 0.67 (0.45, 1.01)  0.61 (0.47, 0.79) | 0.056  <0.001 |
| Smoking status  Never (reference group)  Ex  Current | 0.81 (0.65, 1.01)  0.60 (0.60, 0.96) | 0.065  0.022 | 0.80 (0.65, 0.97)  0.78 (0.63, 0.97) | 0.025  0.024 |
| Weight (kg) | 0.989 (0.985, 0.994) | <0.001 | 0.990 (0.986, 0.994) | <0.001 |
| Skin type^1^ | 1.05 (0.97, 1.12) | 0.19 | 1.04 (0.97, 1.11) | 0.28 |
| Baseline PASI | 1.04 (1.03, 1.05) | <0.001 | 1.04 (1.03, 1.05) | <0.001 |
| Disease duration (years) | 0.995 (0.987, 1.003) | 0.19 | 0.996 (0.989, 1.003) | 0.31 |
| Palms/soles psoriasis | 0.72 (0.58, 0.89) | 0.002 | 0.73 (0.60, 0.90) | 0.003 |
| Chronic plaque size  Small & large (reference group)  Small only  Large only | 0.78 (0.62, 0.99)  0.84 (0.70, 1.01) | 0.037  0.070 | 0.77 (0.61, 0.95)  0.88 (0.74, 1.04) | 0.017  0.16 |
| Hypertension | 0.99 (0.81, 1.22) | 0.95 | 0.96 (0.79, 1.17) | 0.72 |
| Angina | 0.70 (0.40, 1.24) | 0.22 | 0.83 (0.47, 1.45) | 0.51 |
| Diabetes | 0.89 (0.65, 1.22) | 0.47 | 0.87 (0.64, 1.17) | 0.35 |
| Depression | 1.03 (0.84, 1.26) | 0.80 | 1.02 (0.84, 1.24) | 0.83 |
| Other comorbidities | 0.87 (0.74, 1.03) | 0.11 | 0.87 (0.75, 1.02) | 0.095 |

^1^  Results for white ethnicity and skin type are estimated from a model excluding the other due to similarities in these two variables.

**Table S2** Results of sensitivity analyses investigating predictors of PASI 90 at 12 months in patients who initiated adalimumab, etanercept or ustekinumab when 1) patients who withdrew from treatment before the PASI measure were excluded and 2) weighting to account for sampling was applied

|  | **Sensitivity analysis 1** | | **Sensitivity analysis 2** | |
| --- | --- | --- | --- | --- |
| **Predictor** | **Odds ratio (95% CI)** | ***p*-value** | **Odds ratio (95% CI)** | ***p*-value** |
| Biologic therapy  Adalimumab (reference group)  Etanercept  Ustekinumab | 0.21 (0.16, 0.27)  1.31 (1.04, 1.64) | <0.001  0.020 | 0.29 (0.24, 0.36)  1.32 (1.08, 1.62) | <0.001  0.006 |
| Age in years at start of treatment | 0.998 (0.991, 1.006) | 0.68 | 0.997 (0.990, 1.004) | 0.39 |
| Female gender | 0.86 (0.70, 1.05) | 0.14 | 0.80 (0.67, 0.95) | 0.010 |
| White ethnicity | 1.72 (1.24, 2.38) | 0.001 | 1.80 (1.36, 2.38) | <0.001 |
| Work status  In work/student/retired (reference  group)  Unemployed and seeking work  Unemployed due to ill health | 0.69 (0.43, 1.12)  0.76 (0.56, 1.02) | 0.14  0.072 | 0.67 (0.44, 1.01)  0.77 (0.60, 1.00) | 0.054  0.051 |
| Weekly alcohol units | 1.007 (0.998, 1.106) | 0.15 | 1.005 (0.997, 1.012) | 0.23 |
| Smoking status  Never (reference group)  Ex  Current | 0.73 (0.58, 0.92)  0.87 (0.69, 1.10) | 0.008  0.25 | 0.78 (0.64, 0.95)  0.90 (0.73, 1.10) | 0.012  0.30 |
| Weight (kg) | 0.987 (0.983, 0.992) | <0.001 | 0.989 (0.984, 0.992) | <0.001 |
| Baseline PASI | 1.04 (1.02, 1.05) | <0.001 | 1.03 (1.02, 1.05) | <0.001 |
| Nail psoriasis | 0.87 (0.73, 1.05) | 0.15 | 0.86 (0.74, 1.02) | 0.067 |
| Scalp psoriasis | 1.05 (0.85, 1.29) | 0.65 | 1.13 (0.95, 1.35) | 0.18 |
| Palms/soles psoriasis | 0.69 (0.55, 0.87) | 0.002 | 0.73 (0.59, 0.81) | 0.002 |
| Psoriatic arthritis | 1.34 (1.08, 1.66) | 0.007 | 1.23 (1.02, 1.48) | 0.032 |
| Chronic plaque size  Small & large (reference group)  Small only  Large only | 0.79 (0.62, 1.01)  0.84 (0.69, 1.02) | 0.065  0.082 | 0.85 (0.68, 1.05)  0.89 (0.75, 0.06) | 0.14  0.18 |
| Hypertension | 0.90 (0.72, 1.12) | 0.35 | 0.93 (0.77, 1.14) | 0.50 |
| Angina | 0.87 (0.48, 1.59) | 0.65 | 0.86 (0.50, 1.46) | 0.57 |
| Diabetes | 1.00 (0.71, 1.42) | 0.99 | 0.89 (0.76, 1.21) | 0.46 |
| Dyslipidaemia | 0.94 (0.70, 1.26) | 0.70 | 0.98 (0.76, 1.27) | 0.91 |

**Table S3** The baseline characteristics of the biologic-naive cohort

|  | All eligible patients  (*n* = 5885) | All three biologics (*n* = 3079) | Adalimumab  (*n* = 1791) | Etanercept  (*n* = 713) | Ustekinumab  (*n* = 575) |
| --- | --- | --- | --- | --- | --- |
| *Demographics (mean (sd) for continuous variables*, *N (%) for categorical variables)* | | | | | |
| Age (years) | 44.2 (12.9) | 44.2 (13.0) | 43.8 (12.8) | 44.8 (13.0) | 45.0 (13.3) |
| Female sex | 2389 (40.6) | 1250 (40.6) | 752 (42.0) | 283 (39.7) | 215 (37.4) |
| White ethnicity | 5398 (91.7) | 2814 (91.4) | 1642 (91.7) | 655 (91.9) | 517 (89.9) |
| *Social characteristics* |  |  |  |  |  |
| Work status:  In work  Unemployed  and seeking  work  Unemployed  due to ill  health  Student  Retired  Missing (N (%)) | 3755 (63.8)  220 (3.74)  600 (10.2)  136 (2.31)  512 (8.70)  662 (11.3) | 1983 (64.4)  124 (4.04)  348 (11.3)  71 (2.31)  266 (8.64)  287 (9.32) | 1208 (67.5)  68 (3.80)  191 (10.7)  41 (2.29)  146 (8.15)  137 (7.65) | 405 (56.8)  28 (3.93)  78 (10.9)  15 (2.10)  63 (8.84)  124 (17.4) | 370 (64.4)  28 (4.87)  79 (13.7)  15 (2.61)  57 (9.91)  26 (4.52) |
| Weekly alcohol units  Missing (N (%)) | 11.9 (13.5)  2239 (38.1) | 11.7 (13.7)  1127 (36.6) | 11.2 (13.0)  594 (32.2) | 13.1 (14.0)  312 (43.8) | 11.7 (15.5)  221 (38.4) |
| Smoking status  Never  Current  Ex  Missing | 1671 (28.4)  1856 (31.5)  1542 (26.2)  816 (13.9) | 894 (29.0)  999 (32.5)  805 (26.1)  381 (12.4) | 529 (29.5)  589 (32.9)  487 (27.2)  186 (10.4) | 183 (25.7)  198 (27.8)  181 (25.4)  151 (21.2) | 182 (31.7)  212 (36.9)  137 (23.8)  44 (7.65) |
| *Clinical characteristics* | | | | | |
| BMI  Missing (N (%)) | 30.9 (7.17)  406 (6.90) | 31.0 (7.18)  190 (6.17) | 30.9 (7.08)  102 (5.70) | 30.4 (6.60)  50 (7.01) | 32.3 (7.96)  38 (6.61) |
| Weight (kg)  Missing (N (%)) | 90.5 (21.6)  218 (3.70) | 90.8 (21.7)  95 (3.09) | 90.0 (21.0)  50 (2.79) | 89.3 (20.7)  27 (3.79) | 95.4 (24.4)  18 (3.13) |
| Skin type: 6 categories  score^1^  1  2  3  4  5  6  Missing | 783 (13.3)  1713 (29.1)  1746 (29.7)  1071 (18.2)  403 (6.85)  100 (1.70)  69 (1.17) | 411 (13.4)  905 (29.4)  900 (29.2)  550 (17.9)  228 (7.41)  56 (1.82)  29 (0.94) | 238 (13.3)  534 (29.8)  515 (28.8)  319 (17.8)  144 (8.04)  25 (1.40)  16 (0.89) | 109 (15.3)  200 (28.1)  204 (28.6)  121 (17.0)  57 (7.99)  15 (2.10)  7 (0.98) | 64 (11.1)  171 (29.7)  181 (31.5)  110 (19.1)  27 (4.70)  16 (2.78)  6 (1.04) |
| Baseline PASI  Missing (N (%)) | 15.8 (7.79)  672 (11.4) | 15.8 (7.78)  - | 16.0 (7.78)  - | 15.3 (7.58)  - | 15.8 (8.01)  - |
| Disease duration (years)  Missing (N (%)) | 21.6 (12.4)  64 (1.09) | 21.6 (12.5)  31 (1.00) | 21.6 (12.5)  22 (1.23) | 22.4 (12.3)  3 (0.42) | 20.9 (12.6)  6 (1.04) |
| Family history of psoriasis  Missing (N (%)) | 2805 (47.7)  496 (8.43) | 1496 (48.6)  265 (8.61) | 863 (48.2)  149 (8.32) | 353 (49.5)  62 (8.70) | 280 (48.7)  54 (9.39) |
| Flexural psoriasis | 2234 (38.0) | 1297 (42.1) | 763 (42.6) | 302 (42.4) | 232 (40.4) |
| Scalp psoriasis | 4201 (71.4) | 2306 (74.9) | 1357 (7585) | 520 (72.9) | 429 (74.6) |
| Nail psoriasis | 3300 (56.1) | 1785 (58.0) | 1060 (59.2) | 419 (58.8) | 306 (53.2) |
| Palms/soles psoriasis | 1111 (18.9) | 611 (19.8) | 354 (19.8) | 152 (21.3) | 105 (19.8) |
| Chronic plaque small/large  Small only  Large only  Small & large  Missing | 1053 (17.9)  2272 (38.6)  2446 (41.4)  114 (1.94) | 552 (17.9)  1137 (36.9)  1340 (43.5)  50 (1.62) | 322 (18.0)  617 (34.5)  823 (46.0)  29 (1.62) | 136 (19.1)  264 (37.0)  299 (41.9)  14 (1.96) | 94 (16.4)  256 (44.5)  218 (37.9)  7 (1.22) |
| Psoriatic arthritis | 1210 (20.6) | 680 (20.1) | 432 (24.1) | 174 (24.4) | 74 (12.9) |
| Number of comorbidities  0  1–2  3–4  5+ | 1967 (33.4)  3112 (52.9)  718 (12.2)  88 (1.50) | 1010 (32.8)  1631 (53.0)  391 (12.7)  47 (1.53) | 594 (33.2)  971 (54.2)  205 (11.5)  21 (1.17) | 226 (31.7)  374 (52.5)  100 (14.0)  13 (1.82) | 190 (33.5)  286 (49.7)  86 (14.5)  13 (2.30) |
| Number of previous conventional drugs  0  1  2  3+ | 924 (15.7)  1429 (24.3)  1671 (28.4)  1861 (31.6) | 484 (15.7)  768 (24.9)  855 (27.8)  972 (31.6) | 284 (15.9)  426 (23.8)  508 (28.4)  573 (32.0) | 93 (13.0)  183 (25.7)  202 (28.3)  235 (33.0) | 107 (18.6)  159 (27.7)  145 (25.2)  164 (28.5) |
| Baseline concomitant methotrexate | 549 (9.33) | 306 (9.94) | 191 (10.7) | 72 (10.1) | 43 (7.48) |
| Baseline concomitant ciclosporin | 316 (5.37) | 173 (5.62) | 111 (6.20) | 31 (4.35) | 31 (5.39) |

^1^Categories defined as 1: burns easily, never tans, 2: burns easily, tans minimally, 3: burns moderately, tans gradually, 4: burns minimally, tans well, 5: rarely burns, tans profusely, 6: never burns, deeply pigmented.

**Table S4** Response rates (%) for PASI 90, PASI 75 and PASI$\leq$1.5 at 6 and 12 months

|  | Adalimumab | | Etanercept | | Ustekinumab | |
| --- | --- | --- | --- | --- | --- | --- |
|  | 6 months  (n=1791) | 12 months  (n=1853) | 6 months  (n=713) | 12 months  (n=720) | 6 months  (n=575) | 12 months  (n=537) |
| PASI 90 | 50.1 | 49.4 | 20.1 | 22.4 | 49.9 | 53.6 |
| PASI 75 | 71.9 | 70.4 | 45.0 | 49.9 | 74.6 | 76.7 |
| PASI$\leq$1.5 | 51.5 | 51.1 | 19.9 | 23.6 | 52.9 | 55.3 |

**Table S5** Results of the univariate analyses exploring associations between potential risk factors and the attainment of PASI 90 at 6 months in patients who initiated adalimumab, etanercept or ustekinumab

| **Predictor** | **Summary measure** | | **Test statistic** | ***p*-value** | **Unadjusted odds ratio (95% CI)** |
| --- | --- | --- | --- | --- | --- |
|  | **PASI 90: Yes** | **PASI 90: No** |  |  |  |
| Biologic therapy^1^  Adalimumab   Etanercept  Ustekinumab | 67.6  10.8  21.6 | 51.0  32.5  16.4 | 200.9 | <0.001 | 0.25 (0.20, 0.31)  0.99 (0.82, 1.20) |
| Age in years at start of treatment^2^ | 43.9 (12.6) | 44.5 (13.2) | 1.43 | 0.15 | 0.996 (0.991, 1.002) |
| Female gender^1^ | 38.2 | 42.4 | 5.53 | 0.019 | 0.84 (0.73, 0.97) |
| White ethnic group^1^ | 92.1 | 90.9 | 1.43 | 0.23 | 1.17 (0.90, 1.51) |
| Work status^1^  In work  Unemployed  and seeking  work  Unemployed  due to ill  health  Student  Retired | 76.4  3.66  9.83  1.87  8.20 | 66.8  5.06  14.5  3.07  10.6 | 32.4 | <0.001 | 0.63 (0.43, 0.92)  0.59 (0.47, 0.75)  0.53 (0.32, 0.88)  0.68 (0.52, 0.88) |
| Weekly alcohol units^3^ | 8 | 8 | -1.04 | 0.30 | 1.00 (0.99, 1.01) |
| Smoking status^1^  Never  Current  Ex | 35.9  35.3  28.9 | 31.0  38.4  30.6 | 7.18 | 0.028 | 0.79 (0.66, 0.95)  0.81 (0.67, 0.99) |
| BMI^2^ | 30.5 (7.08) | 31.4 (7.24) | 3.33 | <0.001 | 0.982 (0.972, 0.993) |
| Weight^2^ | 89.4 (21.6) | 92.0 (22.0) | 3.76 | 0.001 | 0.994 (0.991, 0.997) |
| Skin type^1^  1  2  3  4  5  6 | 11.6  29.6  30.4  19.3  7.14  1.98 | 14.9  29.7  28.8  17.1  7.73  1.73 | 9.64 | 0.086 | 1.29 (1.02, 1.64)  1.36 (1.07, 1.73)  1.46 (1.13, 1.90)  1.20 (0.86, 1.66)  1.48 (0.84, 2.59) |
| Baseline PASI^3^ | 15.0 | 13.6 | -6.47 | <0.001 | 1.03 (1.02, 1.04) |
| Disease duration (years)^3^ | 20 | 20 | 1.37 | 0.17 | 0.995 (0.989, 1.001) |
| Family history of psoriasis^1^ | 52.2 | 53.9 | 0.86 | 0.35 | 0.93 (0.80, 1.08) |
| Flexural psoriasis^1^ | 43.3 | 41.3 | 1.22 | 0.27 | 1.08 (0.94, 1.25) |
| Scalp psoriasis^1^ | 75.4 | 74.5 | 0.36 | 0.55 | 1.05 (0.89, 1.24) |
| Nail psoriasis^1^ | 57.4 | 58.5 | 0.38 | 0.54 | 0.96 (0.83, 1.10) |
| Palms/soles psoriasis^1^ | 17.4 | 21.7 | 8.70 | 0.003 | 0.76 (0.63, 0.91) |
| Chronic plaque size^1^  Small & large  Small only  Large only | 46.6  16.7  36.7 | 42.4  19.4  38.2 | 6.27 | 0.043 | 0.79 (0.64, 0.96)  0.87 (0.74, 1.02) |
| Psoriatic arthritis^1^ | 22.5 | 21.8 | 0.27 | 0.60 | 1.05 (0.88, 1.24) |
| Hypertension^1^ | 23.6 | 27.3 | 5.40 | 0.020 | 0.82 (0.70, 0.97) |
| Angina^1^ | 1.81 | 2.51 | 1.73 | 0.19 | 0.71 (0.43, 1.18) |
| Heart attack^1^ | 2.08 | 2.03 | 0.01 | 0.92 | 1.03 (0.62, 1.69) |
| Stroke^1^ | 1.13 | 1.26 | 0.10 | 0.75 | 0.90 (0.46, 1.74) |
| Latent TB^1^ | 0.90 | 0.91 | 0.0007 | 0.98 | 0.99 (0.47, 2.10) |
| Diabetes^1^ | 7.08 | 9.93 | 7.71 | 0.006 | 0.69 (0.53, 0.90) |
| Depression^1^ | 21.9 | 24.1 | 2.23 | 0.14 | 0.88 (0.74, 1.04) |
| Dyslipidaemia^1^ | 10.32 | 11.07 | 0.44 | 0.51 | 0.92 (0.73, 1.17) |
| Non-skin cancer^1^ | 0.90 | 1.03 | 0.12 | 0.73 | 0.88 (0.42, 1.83) |
| Other comorbidities^1^ | 44.3 | 48.6 | 5.51 | 0.019 | 0.84 (0.73, 0.97) |
| Number of previous conventional drugs  0  1  2  3+ | 15.7  24.6  27.3  32.4 | 15.8  25.2  28.1  30.9 | 0.80 | 0.85 | 0.98 (0.78, 1.24)  0.97 (0.78, 1.22)  1.05 (0.84, 1.31) |
| Baseline concomitant methotrexate^1^ | 9.72 | 10.1 | 0.12 | 0.73 | 0.96 (0.75, 1.22) |
| Baseline concomitant ciclosporin^1^ | 5.20 | 5.94 | 0.77 | 0.38 | 0.87 (0.64, 1.19) |

^1^ Summary measure: Percentage. Hypothesis test: chi-squared test.
^2^ Summary measure: Mean (standard deviation). Hypothesis test: *t*-test.
^3^ Summary measure: Median. Hypothesis test: Mann Whitney U test.

**Table S6** Results of multivariable logistic regression analyses investigating predictors of PASI 90 at both 6 and 12 months in patients who initiated adalimumab, etanercept or ustekinumab

|  | **PASI 90 6 months** | | **PASI 90 12 months** | |
| --- | --- | --- | --- | --- |
| **Predictor** | **Odds ratio (95% CI)** | ***p*-value** | **Odds ratio (95% CI)** | ***p*-value** |
| Biologic therapy  Adalimumab (reference group)  Etanercept  Ustekinumab | 0.25 (0.20, 0.30)  1.08 (0.89, 1.31) | <0.001  0.43 | 0.29 (0.24, 0.35)  1.32 (1.08, 1.61) | <0.001  0.007 |
| Age at start of treatment | 1.001 (0.994, 1.009) | 0.71 | 0.997 (0.991, 1.004) | 0.43 |
| Female gender | 0.78 (0.66, 0.93) | 0.003 | 0.80 (0.67, 0.95) | 0.011 |
| White ethnicity | 1.48 (1.12, 1.97) | 0.005 | 1.81 (1.36, 2.41) | <0.001 |
| Work status  In work/student/retired (reference  group)  Unemployed and seeking work  Unemployed due to ill health | 0.67 (0.45, 0.99)  0.62 (0.48, 0.82) | 0.068  0.001 | 0.68 (0.45, 1.01)  0.78 (0.60, 1.01) | 0.056  0.064 |
| Weekly alcohol units |  | - | 1.005 (0.997, 1.012) | 0.24 |
| Smoking status  Never (reference group)  Ex  Current | 0.81 (0.66, 0.99)  0.79 (0.63, 0.99) | 0.024  0.019 | 0.76 (0.63, 0.93)  0.89 (0.72, 1.09) | 0.008  0.26 |
| Weight (kg) | 0.990 (0.986, 0.994) | <0.001 | 0.988 (0.984, 0.992) | <0.001 |
| Skin type | 1.04 (0.97, 1.11) | 0.23 |  |  |
| Baseline PASI | 1.04 (1.03, 1.05) | <0.001 | 1.03 (1.02, 1.05) | <0.001 |
| Disease duration | 0.997 (0.99, 1.004) | 0.35 |  |  |
| Palms/soles psoriasis | 0.75 (0.61, 0.91) | 0.004 | 0.73 (0.6, 0.89) | 0.002 |
| Psoriatic arthritis | - | - | 1.22 (1.01, 1.47) | 0.040 |
| Chronic plaque size   Small & large (reference group)  Small only  Large only | 0.78 (0.62, 0.96)  0.87 (0.74, 1.04) | 0.024  0.13 | 0.85 (0.69, 1.06)  0.89 (0.75, 1.05) | 0.14  0.18 |
| Hypertension | 0.93 (0.77, 1.13) | 0.49 | 0.92 (0.75, 1.11) | 0.38 |
| Angina | 0.77 (0.44, 1.33) | 0.34 | 0.82 (0.49, 1.39) | 0.47 |
| Diabetes | 0.89 (0.66, 1.20) | 0.45 | 0.91 (0.68, 1.23) | 0.56 |
| Depression | 1.00 (0.83, 1.21) | 0.99 |  |  |
| Dyslipidaemia | - | - | 0.98 (0.76, 1.27) | 0.88 |
| Other comorbidities | 0.89 (0.76, 1.04) | 0.14 | - | - |
| *Additional analyses with categorised continuous variables* | | | | |
| Age at start of treatment  <30 (reference group)  30-45  45-60  60+ | 1.09 (0.85, 1.39)  1.11 (0.85, 1.46)  0.86 (0.60, 1.21) | 0.51  0.42  0.38 | 0.91 (0.72, 1.16)  0.98 (0.76, 1.27)  0.79 (0.58, 1.10) | 0.46  0.90  0.16 |
| Weekly alcohol units  <14 (reference group)  14+ | - | - | 1.10 (0.90, 1.35) | 0.34 |
| Weight (kg)  <70 (reference group)  70-90  90-110  110+ | 0.68 (0.54, 0.87)  0.56 (0.44, 0.73)  0.46 (0.34, 0.61) | 0.002  <0.001  <0.001 | 0.83 (0.66, 1.05)  0.58 (0.45, 0.74)  0.46 (0.35, 0.62) | 0.12  <0.001  <0.001 |
| Disease duration (years)  <10 (reference group)  10+ | 1.16 (0.94, 1.43) | 0.16 | - | - |

**Table S7** Calibration and discrimination analysis statistics for the multivariable regression models

|  | **PASI 90, 6 months** | **PASI 90, 12 months** | **PASI 75, 6 months** | **PASI 75, 12 months** | **PASI**$\boldsymbol{\leq}$**1.5, 6 months** | **PASI**$\boldsymbol{\leq}$**1.5, 12 months** |
| --- | --- | --- | --- | --- | --- | --- |
| ***Calibration*** | | | | | | |
| **Estimated probability** | **Percentage who observed the outcome** | | | | | |
| $\hat{p}<$0.05 | 0%^1^ | -^3^ | -^3^ | 85.7% | -^3^ | 75.0% |
| 0.05$\leq\hat{p}<$0.10 | 20.7% | -^3^ | 16.7% | 18.2% | -^3^ | 0% |
| 0.10$\leq\hat{p}<$0.15 | 12.4% | 0%^1^ | 12.5% | 20.6% | -^3^ | 19.0% |
| 0.15$\leq\hat{p}<$0.20 | 17.7% | 0%^2^ | 17.5% | 18.3% | 33.3% | 18.8% |
| 0.20$\leq\hat{p}<$0.25 | 21.5% | 23.5% | 22.9% | 20.2% | 33.3% | 22.7% |
| 0.25$\leq\hat{p}<$0.30 | 22.1% | 38.5% | 24.2% | 25.9% | 45.5% | 25.9% |
| 0.30$\leq\hat{p}<$0.35 | 32.6% | 40.5% | 31.9% | 30.9% | 38.5% | 27.1% |
| 0.35$\leq\hat{p}<$0.40 | 40.2% | 30.2% | 40.3% | 37.1% | 36.3% | 42.7% |
| 0.40$\leq\hat{p}<$0.45 | 40.5% | 40.0% | 40.1% | 40.7% | 32.8% | 42.1% |
| 0.45$\leq\hat{p}<$0.50 | 48.9% | 43.1% | 48.3% | 42.7% | 47.2% | 46.2% |
| 0.50$\leq\hat{p}<$0.55 | 51.4% | 55.1% | 51.9% | 57.8% | 53.1% | 50.9% |
| 0.55$\leq\hat{p}<$0.60 | 57.3% | 58.2% | 59.3% | 58.1% | 59.7% | 59.0% |
| 0.60$\leq\hat{p}<$0.65 | 61.0% | 57.9% | 60.1% | 63.4% | 61.0% | 64.3% |
| 0.65$\leq\hat{p}<$0.70 | 72.0% | 68.5% | 66.9% | 69.1% | 65.9% | 65.3% |
| 0.70$\leq\hat{p}<$0.75 | 72.0% | 75.5% | 85.7% | 69.2% | 75.1% | 73.1% |
| 0.75$\leq\hat{p}<$0.80 | 80.0% | 77.0% | 85.7% | 81.0% | 80.2% | 100%^1^ |
| 0.80$\leq\hat{p}<$0.85 | 100% | 83.3% | 85.7% | 100% | 80.5% | -^3^ |
| 0.85$\leq\hat{p}<$0.90 | 100%^1^ | 87.3% | 85.7% | 50.0% | 85.1% | -^3^ |
| 0.90$\leq\hat{p}<$0.95 | 100%^1^ | 86.7% | 85.7% | 50.0% | 87.5% | -^3^ |
| 0.95$\leq\hat{p}<$1 | -^3^ | 75.0% | 85.7% | 50.0% | 75.0% | -^3^ |
| Hosmer – Lemeshow test *p*-value^4^ | 0.606 | 0.479 | 0.159 | 0.787 | 0.911 | 0.500 |
| ***Discrimination: AUC (95% CI)*** ^5^ | | | | | | |
|  | 0.6926 (0.6741, 0.7111) | 0.6897 (0.6712, 0.7083) | 0.6976 (0.6779, 0.7173) | 0.6782 (0.6581, 0.6983) | 0.6831 (0.6645, 0.7017) | 0.6777 (0.6591, 0.6964) |

^1^ 1 observation, ^2^ 2 observations, ^3^ no observations, ^4^ A significant result indicates poor fit to the data
^5^Intrepeted as the probability that, given one randomly selected patient who did achieve the outcome, and one randomly selected patient who did not achieve the outcome, the patient who observed the outcome has a higher predicted probability of the outcome that the patient who did not observe the outcome.

**Table S8** Estimated risk differences and Number Needed to Treats (NNTs) for each predictor included in the multivariable analysis on the attainment of PASI 90 at 6 months in patients who initiated adalimumab, etanercept or ustekinumab

| Predictor^1^ | Risk (95% CI) | Risk in reference group (95% CI) | Risk difference (95% CI) | NNT (95% CI) ^2^ |
| --- | --- | --- | --- | --- |
| Biological therapy  Adalimumab (reference group)  Etanercept    Ustekinumab | 0.204 (0.174, 0.233)  0.513 (0.473, 0.553) | 0.495 (0.472, 0.517) | -0.291 (-0.328, -0.254)  0.019 (-0.028, 0.065) | -3.4 ( -3.9, -3.0)  53.7 |
| Age in years at start of treatment: 40 years | 0.440 (0.418, 0.461) | 0.416 (0.388, 0.445) | 0.023 (-0.015, 0.061) | 43.3 |
| Female gender | 0.399 (0.372, 0.426) | 0.453 (0.431, 0.475) | -0.054 (-0.090,  -0.018) | -18.4 (-55.3,  -11.1) |
| White ethnicity | 0.439 (0.421, 0.456) | 0.352 (0.297, 0.408) | 0.088 (0.027, 0.150) | 11.3 (6.66, 37.5) |
| Work status  In  work/student/retired  (reference group)  Unemployed and  seeking work  Unemployed due to  ill health | 0.367 (0.286, 0.448)  0.348 (0.298, 0.399) | 0.447 (0.428, 0.465) | -0.080 (-0.163, 0.004)  -0.099 (-0.154,  -0.043) | 12.5  -10.1 (-23.1,  -6.5) |
| Smoking status  Never (reference  group)  Ex    Current | 0.416 (0.387, 0.445)  0.411 (0.378, 0.443) | 0.466 (0.435, 0.496) | -0.050 (-0.093, -0.007)  -0.055 (-0.100, -0.009) | -20.0 (-150,  -10.7)  -18.3 (-109,  -9.96) |
| Weight: 90 kg | 0.391 (0.367, 0.416) | 0.468 (0.444, 0.492) | -0.076 (-0.112,  -0.041) | -13.1 (-24.7,  -8.9) |
| Skin type: categories 4–6 vs. 1–3 | 0.446 (0.413, 0.478) | 0.425 (0.406, 0.445) | 0.020 (-0.017, 0.060) | 49.5 |
| Baseline PASI: 15 | 0.486 (0.461, 0.512) | 0.386 (0.364, 0.408) | 0.099 (0.066, 0.134) | 10.1 (7.5, 15.2) |
| Disease duration: 10 years | 0.437 (0.418, 0.455) | 0.404 (0.363, 0.444) | 0.033 (-0.012, 0.079) | 29.9 |
| Palms/soles psoriasis | 0.380 (0.343, 0.418) | 0.443 (0.425, 0.462) | -0.063 (-0.106,  -0.021) | -15.8 (-48.4,  -9.41) |
| Chronic plaque size  Small & large  (reference group)  Small only    Large only | 0.398 (0.358, 0.437)  0.423 (0.395, 0.450) | 0.452 (0.427, 0.477) | -0.054 (-0.106,  -0.007)  -0.029 (-0.066, 0.009) | -18.5 (-134,  -9.93)  -34.7 |
| Hypertension | 0.420 (0.384, 0.456) | 0.435 (0.415, 0.454) | -0.015 (-0.057, 0.028) | -67.4 |
| Angina | 0.375 (0.261, 0.489) | 0.432 (0.416, 0.449) | -0.058 (-0.178, 0.062) | -17.3 |
| Diabetes | 0.408 (0.347, 0.469) | 0.433 (0.416, 0.450) | -0.025 (-0.090, 0.040) | -40.2 |
| Depression | 0.431 (0.395, 0.467) | 0.431 (0.412, 0.450) | -0.00006  (-0.042, 0.042) | -16987 |
| Other comorbidities | 0.417 (0.392, 0.442) | 0.443 (0.420, 0.466) | -0.026 (-0.061, 0.008) | -38.1 |

^1^ Continuous variables are dichotomised at the median

^2^ 95% confidence intervals are only shown for NNTs where the corresponding risk difference confidence interval did not contain 0.

**Table S9** Results of multivariable logistic regression analyses investigating predictors of PASI 75 at both 6 and 12 months in patients who initiated adalimumab, etanercept or ustekinumab

|  | **PASI 75 6 months** | | **PASI 75 12 months** | |
| --- | --- | --- | --- | --- |
| **Predictor** | **Odds ratio (95% CI)** | ***p*-value** | **Odds ratio (95% CI)** | ***p*-value** |
| Biologic therapy  Adalimumab (reference group)  Etanercept  Ustekinumab | 0.31 (0.26, 0.37)  1.24 (1.00, 1.55) | <0.001  0.055 | 0.41 (0.34, 0.49)  1.51 (1.20, 1.91) | <0.001  <0.001 |
| Female gender | 0.69 (0.58, 0.82) | <0.001 | 0.76 (0.63, 0.91) | 0.002 |
| White ethnicity | 1.46 (1.10, 1.95) | 0.010 | 1.77 (1.33, 2.34) | <0.001 |
| Work status  In work/student/retired (reference  group)  Unemployed and seeking work  Unemployed due to ill health | 0.62 (0.42, 0.93)  0.61 (0.47, 0.79) | 0.019  <0.001 | 0.63 (0.43, 0.93)  0.74 (0.57, 0.97) | 0.019  0.028 |
| Weekly alcohol units |  |  | 1.001 (0.993, 1.009) | 0.75 |
| Smoking status  Never (reference group)  Ex  Current | 1.01 (0.82, 1.24)  0.93 (0.74, 1.16) | 0.93  0.52 | 0.88 (0.72, 1.07) 0.92 (0.73, 1.14) | 0.20  0.44 |
| Weight (kg) | 0.988 (0.984, 0.992) | <0.001 | 0.986 (0.982, 0.990) | <0.001 |
| Baseline PASI | 1.05 (1.04, 1.06) | <0.001 | 1.05 (1.03, 1.06) | <0.001 |
| Palms/soles psoriasis | 0.71 (0.59, 0.87) | 0.001 | 0.82 (0.67, 1.01) | 0.060 |
| Chronic plaque size   Small & large (reference group)  Small only  Large only | 0.99 (0.79, 1.24)  1.01 (0.84, 1.21) | 0.92  0.92 | 0.95 (0.76, 1.19)  0.97 (0.81, 1.15) | 0.67  0.71 |
| Hypertension | 0.92 (0.76, 1.11) | 0.36 | - | - |
| Latent TB | 0.53 (0.24, 1.18) | 0.12 | - | - |
| Diabetes | 0.98 (0.73, 1.30) | 0.87 | - | - |
| Depression | 0.93 (0.77, 1.13) | 0.49 | 0.91 (0.75, 1.11) | 0.34 |
| Baseline ciclosporin therapy | 0.68 (0.49, 0.95) | 0.023 | - | - |
| Number of previous conventional drugs  0 (reference group)  1  2  3+ |  |  | 1.06 (0.82, 1.36)  1.12 (0.87, 1.43)  1.24 (0.97, 1.58) | 0.68  0.38  0.087 |

The results for PASI 75 indicated that female gender, unemployment (both those seeking work and those unemployed due to ill health), a higher weight and psoriasis involving the palms and/or soles were associated with reduced odds of achieving this outcome at both 6 and 12 months, and that white ethnicity was associated with increased odds of achieving PASI 75 at both time points. Baseline ciclosporin therapy was also associated with this outcome at 6 months.

**Table S10** Results of multivariable logistic regression analyses investigating predictors of absolute PASI$\leq$1.5 at both 6 and 12 months in patients who initiated adalimumab, etanercept or ustekinumab

|  | **PASI** $\boldsymbol{\leq}$**1.5 6 months** | | **PASI** $\boldsymbol{\leq}$**1.5 12 months** | |
| --- | --- | --- | --- | --- |
| **Predictor** | **Odds ratio (95% CI)** | ***p*-value** | **Odds ratio (95% CI)** | ***p*-value** |
| Biologic therapy  Adalimumab (reference group)  Etanercept  Ustekinumab | 0.23 (0.18, 0.28)  1.13 (0.93, 1.38) | <0.001  0.21 | 0.28 (0.23, 0.34)  1.32 (1.08, 1.61) | <0.001  0.007 |
| Age in years at start of treatment | 1.000 (0.993, 1.006) | 0.90 | 0.999 (0.992, 1.005) | 0.70 |
| Female gender | 0.81 (0.69, 0.95) | 0.012 | 0.79 (0.67, 0.93) | 0.004 |
| White ethnicity^1^ | 1.34 (1.02, 1.77) | 0.037 | 1.82 (1.38, 2.41) | <0.001 |
| Work status  In work/student/retired (reference  group)  Unemployed and seeking work  Unemployed due to ill health | 0.79 (0.54, 1.16)  0.64 (0.50, 0.82) | 0.23  0.001 | 0.64 (0.43, 0.95)  0.83 (0.64, 1.07) | 0.028  0.14 |
| Smoking status  Never (reference group)  Ex  Current | 0.76 (0.63, 0.93)  0.77 (0.62, 0.96) | 0.006  0.018 | 0.80 (0.66, 0.96)  0.83 (0.67, 1.02) | 0.020  0.070 |
| Weight (kg) | 0.991 (0.987, 0.995) | <0.001 | 0.988 (0.984, 0.992) | <0.001 |
| Skin type^1^ | 1.04 (0.98, 1.12) | 0.15 | - | - |
| Baseline PASI | 0.99 (0.98, 1.00) | 0.010 | 0.99 (0.98, 1.00) | 0.010 |
| Nail psoriasis | - | - | 0.89 (0.76, 1.04) | 0.15 |
| Palms/soles psoriasis | 0.82 (0.68, 1.00) | 0.049 | 0.72 (0.59, 0.88) | 0.001 |
| Chronic plaque size  Small & large (reference group)  Small only  Large only | 0.82 (0.66, 1.02)  0.92 (0.78, 1.09) | 0.068  0.35 | 0.89 (0.72, 1.11)  0.93 (0.78, 1.10) | 0.30  0.37 |
| Hypertension | 0.96 (0.79, 1.17) | 0.71 | 0.91 (0.75, 1.10) | 0.32 |
| Angina |  |  | 0.78 (0.46, 1.31) | 0.35 |
| Diabetes | 0.82 (0.61, 1.10) | 0.18 | 0.87 (0.65, 1.17) | 0.37 |
| Dyslipidaemia |  |  | 0.93 (0.72, 1.20) | 0.58 |
| Other comorbidities | 0.90 (0.77, 1.05) | 0.19 | - | - |
| Baseline ciclosporin therapy |  |  | 1.15 (0.84, 1.57) | 0.38 |

^1^  Results for white ethnicity and skin type are estimated from a model excluding the other due to similarities in these two variables.

As expected, findings for absolute PASI$\leq$1.5 were similar to those for PASI 90, with comparable effect sizes for the association between gender, white ethnicity, smoking status (both ex and current), psoriasis involving the palms and/or soles, plaque size at both time points, unemployment due to ill heath at 6 months and nail psoriasis at 12 months. An association with baseline PASI was less apparent.

**Table S11** Results of interaction analyses investigating differential relative and absolute effects of each predictor on the attainment of PASI 90 at 6 months across patients who initiated adalimumab, etanercept or ustekinumab

|  | Interaction of odds ratios^1^ | | Interaction of risk differences^2^ | |
| --- | --- | --- | --- | --- |
| Predictor^3^ | Interaction  (95% CI) | *p*-value | Interaction  (95% CI) | *p*-value |
| Age in years at start of treatment: 40 years  Etanercept  Ustekinumab | 1.07 (0.69, 1.68)  1.06 (0.71, 1.59) | 0.75  0.78 | -0.002 (-0.081, 0.078)  0.013 (-0.087, 0.114) | 0.96  0.79 |
| Female gender  Etanercept  Ustekinumab | 1.40 (0.92, 2.15)  1.04 (0.70, 1.55) | 0.12  0.86 | 0.079 (0.002, 0.155)  0.009 (-0.088, 0.105) | 0.044*  0.85 |
| White ethnicity^4^  Etanercept  Ustekinumab | 0.70 (0.33, 1.47)  1.24 (0.63, 2.42) | 0.34  0.53 | -0.090 (-0.219, 0.040)  0.048 (-0.104, 0.201) | 0.18  0.54 |
| Work status: Unemployed and seeking work vs. in work/student/retired  Etanercept  Ustekinumab | 0.54 (0.14, 2.04)  0.75 (0.29, 1.92) | 0.36  0.54 | -0.048 (-0.215, 0.119)  -0.068 (-0.288, 0.151) | 0.58  0.54 |
| Work status: Unemployed due to ill health vs. in work/student/retired  Etanercept  Ustekinumab | 1.06 (0.51, 2.20)  1.21 (0.67, 2.19) | 0.87  0.52 | 0.060 (-0.053, 0.172)  0.044 (-0.096, 0.185) | 0.30  0.53 |
| Smoking status: ex vs. never  Etanercept  Ustekinumab | 1.29 (0.75, 2.22)  1.08 (0.68, 1.73) | 0.35  0.73 | 0.066 (-0.034, 0.165)  0.019 (-0.094, 0.133) | 0.20  0.74 |
| Smoking status: current vs. never  Etanercept  Ustekinumab | 0.99 (0.56, 1.74)  1.32 (0.79, 2.22) | 0.97  0.29 | 0.027 (-0.075, 0.129)  0.067 (-0.058, 0.192) | 0.61  0.29 |
| Weight: 90 kg  Etanercept  Ustekinumab | 1.01 (0.66, 1.56)  1.16 (0.78, 1.72) | 0.95  0.46 | 0.035 (-0.041, 0.112)  0.035 (-0.060, 0.130) | 0.37  0.47 |
| Skin type: categories 4–6 vs. 1–3^1^  Etanercept  Ustekinumab | 1.47 (0.94, 2.32)  1.09 (0.71, 1.68) | 0.092  0.70 | 0.065 (-0.019, 0.149)  0.020 (-0.082, 0.124) | 0.13  0.70 |
| Baseline PASI: 15  Etanercept  Ustekinumab | 0.97 (0.64, 1.48)  0.97 (0.66, 1.43) | 0.89  0.88 | -0.042 (-0.119, 0.035)  -0.008 (-0.101, 0.085) | 0.28  0.87 |
| Disease duration: 10 years  Etanercept  Ustekinumab | 0.63 (0.36, 1.12)  0.78 (0.48, 1.27) | 0.12  0.32 | -0.095 (-0.199, 0.010)  -0.058 (-0.175, 0.059) | 0.077  0.33 |
| Palms/soles psoriasis  Etanercept  Ustekinumab | 0.81 (0.46, 1.40)  0.93 (0.56, 1.54) | 0.45  0.78 | -0.008 (-0.094, 0.079)  -0.017 (-0.136, 0.102) | 0.87  0.78 |
| Chronic plaque size: small only vs. small & large  Etanercept  Ustekinumab | 1.32 (0.76, 2.34)  1.65 (0.94, 2.90) | 0.32  0.081 | 0.074 (-0.030, 0.178)  0.120 (-0.017, 0.254) | 0.16  0.081 |
| Chronic plaque size: large only vs. small & large  Etanercept  Ustekinumab | 0.66 (0.40, 1.07)  0.89 (0.58, 1.37) | 0.088  0.61 | -0.060 (-0.143, 0.022)  -0.027 (-0.129, 0.076) | 0.15  0.61 |
| Hypertension  Etanercept  Ustekinumab | 1.12 (0.70, 1.79)  0.97 (0.62, 1.51) | 0.64  0.90 | 0.024 (-0.063, 0.111)  -0.007 (-0.116, 0.103) | 0.58  0.90 |
| Angina  Etanercept  Ustekinumab | 2.06 (0.45, 9.34)  3.09 (0.93, 10.26) | 0.35  0.066 | 0.169 (-0.087, 0.426)  0.260 (-0.010, 0.530) | 0.20  0.059 |
| Diabetes  Etanercept  Ustekinumab | 1.16 (0.56, 2.44)  0.79 (0.41, 1.53) | 0.69  0.49 | 0.030 (-0.108, 0.169)  -0.055 (-0.213, 0.104) | 0.67  0.50 |
| Depression  Etanercept  Ustekinumab | 1.49 (0.90, 2.47)  0.79 (0.50, 1.25) | 0.12  0.32 | 0.07 (-0.029, 0.163)  -0.056 (-0.167, 0.055) | 0.17  0.33 |
| Other comorbidities  Etanercept  Ustekinumab | 1.17 (0.77, 1.79)  0.98 (0.67, 1.45) | 0.46  0.93 | 0.036 (-0.038, 0.112)  -0.004 (-0.098, 0.090) | 0.34  0.93 |

^1^ Defined as the ratio of odds ratios which compares the odds ratio ($OR$) in patients who received etanercept/ustekinumab vs. adalimumab across the specified subgroups.

^2^ Defined as the difference in risk differences which is the absolute difference in risk differences in patients who received etanercept/ustekinumab vs. adalimumab across the specified subgroups.

^3^ Continuous variables are dichotomised at the median

^4^ Results for white ethnicity and skin type are estimated from a model excluding the other due to similarities in these two variables. ^*^ *P* < 0.05.
